# Theoretical analyses on orbital angular momentum modes in conventional graded-index multimode fibre

**DOI:** 10.1038/s41598-017-04380-7

**Published:** 2017-06-21

**Authors:** Shi Chen, Jian Wang

**Affiliations:** 1 Wuhan National Laboratory for Optoelectronics, School of Optical and Electronic Information, Huazhong University of Science and Technology, Wuhan, 430074 Hubei China; 2 Shenzhen Institute of Huazhong University of Science and Technology, Shenzhen, 518000 China

## Abstract

Orbital angular momentum (OAM) modes are another mode basis to represent spatial modes. There have been increasing interests in exploiting OAM modes in specialty fibres. In this paper, we present a comprehensive characterisation of OAM modes in conventional graded-index multimode fibre (MMF). 1) We synthesise the circularly polarized OAM modes by properly combining two fold degenerate cylindrical vector modes (eigenmodes) and analyse the total angular momentum, i.e. spin angular momentum and orbital angular momentum. 2) We divide all the OAM modes of the conventional graded-index MMF into 10 OAM mode groups with effective refractive index differences between different mode groups above 10^−4^ enabling low-level inter-group crosstalk. 3) We study the chromatic dispersion, differential group delay, effective mode area, and nonlinearity for each OAM mode group over a wide wavelength ranging from 1520 to 1630 nm covering the whole C band and L band. 4) We discuss the performance tolerance to fibre ellipticity and bending. 5) We further address the robustness of performance against fibre perturbations including the core size, index contrast and the imperfect index profile of the practically fabricated MMFs. The obtained results may provide theoretical basis for further space-division multiplexing applications employing OAM modes in conventional graded-index MMF.

## Introduction

Since the first deployment of fibre-optic communication systems three decades ago, the transmission capacity of single-mode fibres (SMFs) has experienced a tremendous growth by four orders of magnitude^1^. However, with the SMF capacity rapidly approaching the Shannon limit due to the fibre Kerr nonlinearity, the ever-increasing capacity demand will keep 2 to 3 orders of magnitude in excess of the capacity of SMFs^2^. Fibre-optic communications are all about the exploitation of different physical dimensions of light waves, such as complex amplitude, frequency (or wavelength), time and polarization^3^. Conventional techniques in fibre-optic communications including wavelength-division multiplexing (WDM), time-division multiplexing (TDM) and polarization-division multiplexing (PDM) have almost reached their scalability limits^4^. To overcome the emerging capacity crunch, space-division multiplexing (SDM) exploiting the space domain (the only known physical dimension of light waves left) seems the next promising solution to further scale the transmission capacity and spectral efficiency of fibre-optic communication systems^4–6^. As one important subset of SDM employing the spatial modes, mode-division multiplexing (MDM) over multi-mode fibres (MMFs) has recently under intense investigation. So far there have been lots of MDM transmission experiments using orthogonal modes over MMFs that support only a small number of modes, also called few-mode fibres (FMFs), to promote the transmission capacity and spectral efficiency^7^. Besides, it has proven to be challenging to realise MDM transmission in conventional MMFs supporting hundreds of modes^8^. Considering that nowadays MMFs are widespread in local and campus-area networks because of their significantly lower costs for transceivers, connectors, and connector installation while meeting and exceeding the requested bandwidth and reliability of the most demanding networks, MDM transmission in MMFs deserves our special attention.

Generally, spatial linearly polarized (LP) modes are adopted in FMF and MMF for MDM to transmit independent data streams. LP modes can be regarded as the proper linear combination of eigenmodes guided by the FMF and MMF. Very recently, the modulation and multiplexing of orbital angular momentum (OAM) states of light in communication systems have gained much interest^9–12^, both in free-space optical communications^13–19^ and fibre-optic transmission systems^20–30^. OAM-carrying light beams are characterized by a helical phase front of exp(*ilφ*), possessing an OAM of *lħ* per photon, where *l* is the topological charge number, *φ* corresponds to the azimuthal angle, and *ħ* is the reduced Planck constant^31, 32^. In principle, *l* can take arbitrary integer number ranging from −∞ to +∞, and OAM modes with different *l* values are intrinsic orthogonal. Similar to other mode bases in free space or fibre^33–35^, OAM modes are another mode basis with which to represent spatial modes. One can use different mode bases for MDM, and so does OAM modes. OAM modes in fibre can be also regarded as the proper linear combination of fibre eigenmodes. Remarkably, different types of specialty fibres could be used to support OAM modes, including vortex fibre with high-refractive-index ring^20, 36^, multi-OAM multi-ring fibre^37, 38^, photonic crystal fibre^39^, supermode OAM fibre^40^, air-core fibre^41^, and inverse-parabolic graded-index fibre^42^. Actually, FMF can also support OAM modes and several experimental works on OAM modes transmission and multiplexing through FMF have been reported^22–28^. Note that only a few low-order OAM modes are supported in FMF. In this scenario, considering the fact that conventional MMF can support hundreds of eigenmodes and the similarity between the FMF and conventional MMF, one would expect to see whether conventional MMF can support OAM modes and it is meaningful to study the characteristics of OAM modes in conventional MMF.

In this paper, we present a comprehensive characterisation of OAM modes in conventional graded-index MMF (e.g. OM3 fibre). We simulate and analyse in detail the properties of OAM modes in an idealized conventional graded-index MMF with square law profile. The MMF has a 50-µm core diameter and a 125-µm cladding diameter. The relative refractive index difference between the core center and cladding is 1% at 632.8 nm, which supports 10 mode groups at 1550 nm. Firstly, we study all cylindrical vector mode (eigenmode) groups and then OAM mode groups that can be supported in the MMF. The effective refractive index (n_eff_) differences between different mode groups can be maintained above 10^−4^, which enables low inter-group crosstalk^43^. Secondly, we calculate the chromatic dispersion (*D*_*λ*_), differential group delay (DGD), effective mode area (A_eff_), and nonlinearity (γ) for each OAM mode group over a wide wavelength ranging from 1520 nm to 1630 nm covering the whole C band and L band (1530 to 1625 nm). Thirdly, we investigate the impacts of fibre ellipticity and fibre bend radius on the performance of OAM modes. Fourthly, we discuss the robustness of performance against fibre perturbations including the core size, index contrast between the core and cladding and the imperfect index profile of the real MMFs. Finally, the characterisation work is summarized at the end.

## Results

### Cylindrical vector modes in conventional graded-index MMF

**Figure 1:**
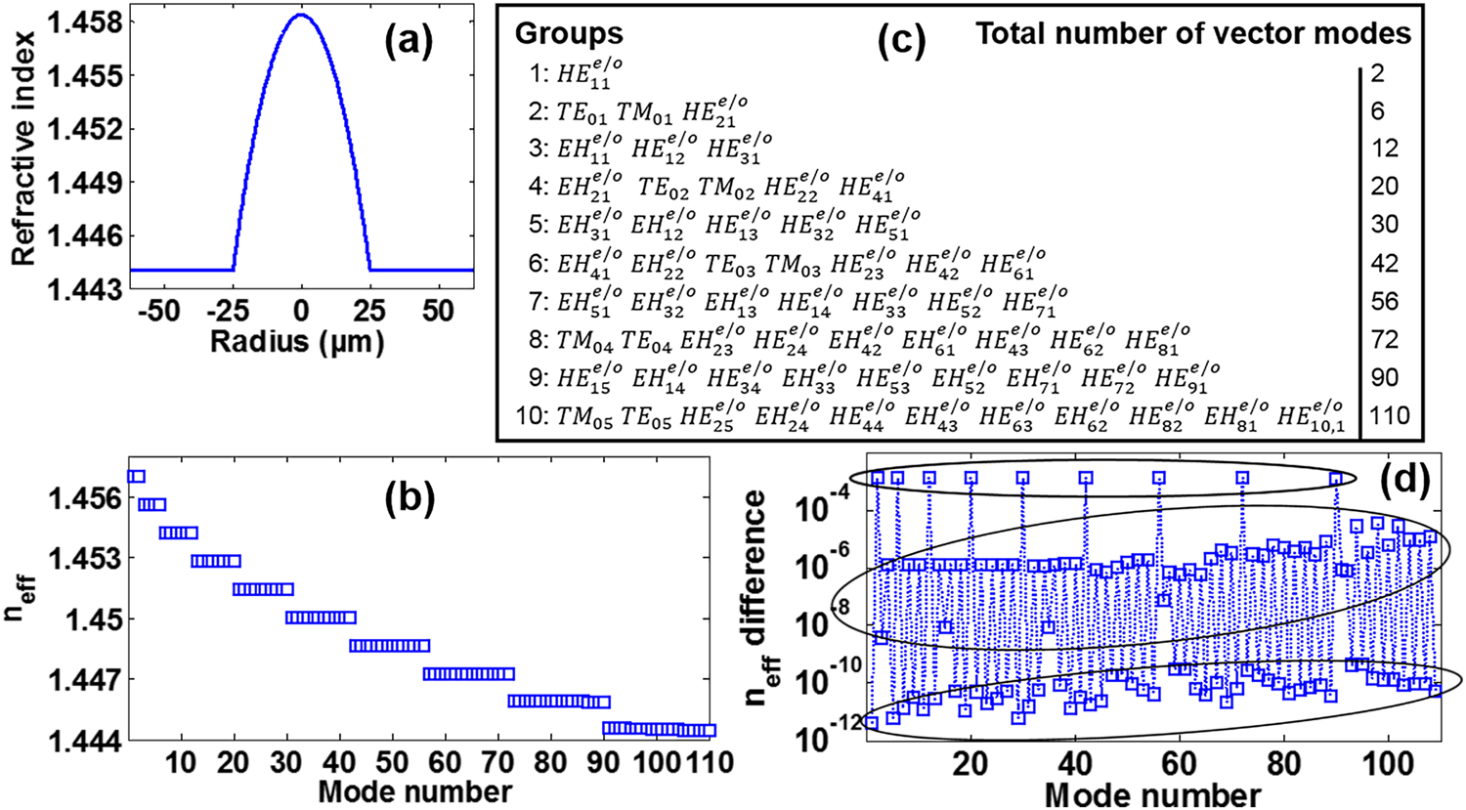
(**a**) Index profile of an idealized conventional graded-index MMF (e.g. OM3) with parameters: r_core_ = 25 μm, *n*_1_ = 1.458369, *n*_2_ = 1.4440 at 1550 nm. (**b**) n_eff_ of all 110 fibre cylindrical vector modes (eigenmodes) at 1550 nm. (**c**) 10 mode groups in conventional graded-index MMF at 1550 nm. Each $H{E}_{mn}^{e/o}$ *or* $E{H}_{mn}^{e/o}$ mode is two fold degenerate, comprising even and odd modes. (**d**) n_eff_ differences in two adjacent vector modes. Top elliptical circle: n_eff_ differences between different mode groups. Middle elliptical circle: n_eff_ differences between different HE/EH/TE/TM modes in the same groups. Bottom elliptical circle: n_eff_ differences between the two fold degenerate modes.

In this work the MMF we study is an idealized conventional circular core optical fibre with a graded-index profile, e.g. an OM3 fibre. The radii of the fibre core and cladding are r_core_ = 25 μm and r_cladding_ = 62.5 μm, respectively. The relative refractive index difference (Δ = (*n*_1_ − *n*_2_)/*n*_2_) measured between the fibre core center (*n*_1_) and cladding (*n*_2_) is Δ = 1% at 632.8 nm. According to the three-term Sellmeier equation^44^, we can theoretically get the fibre index profile at 1550 nm with *GeO*_2_-doped core index *n*_1_ = 1.458369 and pure-*SiO*_2_ cladding index *n*_2_ = 1.4440, as shown in Fig. 1(a). Figure 1(b) shows the n_eff_ of all 110 fibre cylindrical vector modes at 1550 nm calculated by using a full-vector finite-element mode solver. Obviously, these 110 eigenmodes can be divided into 10 mode groups according to the different n_eff_ values. Specifically, the n_eff_ values in the same mode group are approximate equal, while different mode groups possess relatively large n_eff_ difference. Each mode group contains a set of two fold strictly degenerate modes as listed in Fig. 1(c). That is, each ${{\rm{HE}}}_{mn}^{e/o}$ or ${{\rm{EH}}}_{mn}^{e/o}$ comprises even and odd modes, hence 110 fibre eigenmodes are supported in total. Figure 1(d) shows the n_eff_ differences in arbitrary two adjacent vector modes. Remarkably, n_eff_ differences can be divided into three types as marked with black elliptical circles. One can see that n_eff_ differences between different mode groups in the top elliptical circle remain above 10^−4^, which benefits negligible inter-group crosstalk. n_eff_ differences between the two fold degenerate ${{\rm{HE}}}_{mn}^{e/o}$ and ${{\rm{EH}}}_{mn}^{e/o}$ modes are in the bottom elliptical circle. n_eff_ differences between different HE/EH/TE/TM modes in the same groups are in the middle elliptical circle.

### OAM modes in conventional graded-index MMF

Remarkably, OAM-carrying light beam possesses a total angular momentum (AM) of (*l* + *s*) *ħ* per photon, where the OAM values *lħ*, and the spin angular momentum (SAM) valuing *sħ* is associated with circular polarization with *s* = ±1 corresponding to left- or right-circular polarization, respectively^45^. In this work, OAM modes are denoted by $OA{M}_{\pm l,m}^{L/R}$. *l* and *m* in the subscript refer to azimuthal and radial indices, where *l* is the topological charge number, and *m* is the number of nulls radially in the intensity profile of the OAM mode. R and L in the superscript represent right and left handed circular polarization with SAM of −*ħ* and *ħ*, respectively. The total number of supported circular OAM modes contains all degeneracies in circular polarizations and in rotation directions of the phase front of the fields. The circularly polarized OAM modes in the fibre can be obtained by properly combining even and odd modes of *HE*_*nm*_ or *EH*_*mn*_ modes with a ±π/2 phase shift, i.e. $H{E}_{lm}^{even}\pm i\ast H{E}_{lm}^{odd}$ and $E{H}_{lm}^{even}\pm i\ast E{H}_{lm}^{odd}$ and the corresponding topological charge number is ±(*l* − 1) and ±(*l* + 1), respectively. For $H{E}_{lm}^{even}\pm i\ast H{E}_{lm}^{odd}$ combination, the circular polarization and phase front of the OAM mode rotate in the same directions, while $E{H}_{lm}^{even}\pm i\ast E{H}_{lm}^{odd}$ with a circular polarization in the opposite direction with respect to the field phase front rotation. In particular, the sum of *TE*_0*m*_ and *TM*_0*m*_ modes with a ±π/2 phase shift carries the same magnitude of SAM and OAM, while the rotation direction of the circular polarization and phase front is opposite, thus having opposite signs in the SAM and OAM, making the total AM equal to zero^46^.

**Figure 2:**
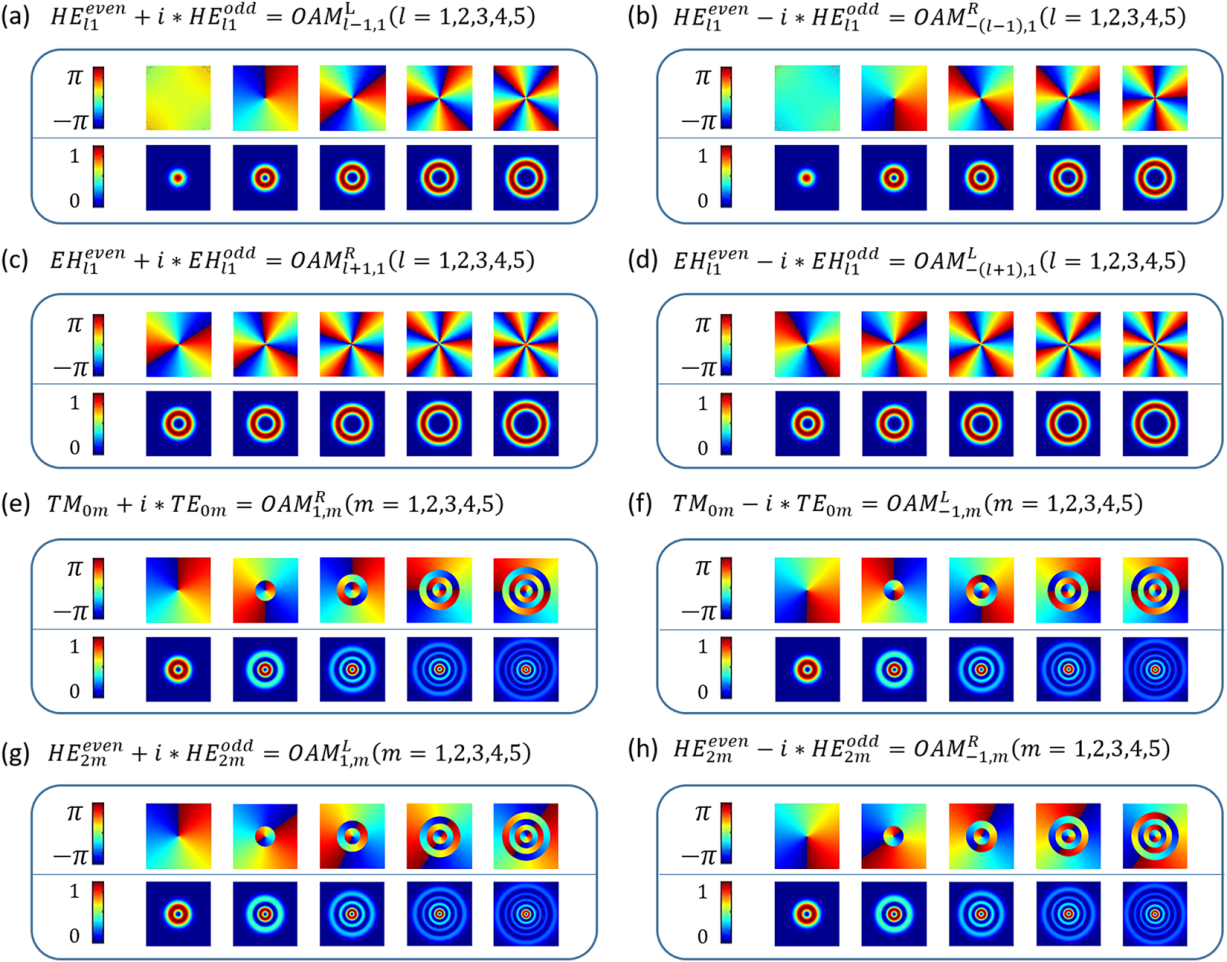
Part of spatial phase distributions of the x-component electric field and intensity profiles of the OAM modes in conventional graded-index MMF.

Figure 2 depicts part of calculated spatial phase distributions of the x-component electric field and intensity profiles of the OAM modes. $H{E}_{1m}^{even}\pm i\ast H{E}_{1m}^{odd}$ features almost constant phase distributions of 0 with the topological charge number equal to zero, and we regard them as *OAM*_0,*m*_. In particular, one can clearly see the spiral phase distributions of $H{E}_{lm}^{even}\pm i\ast H{E}_{lm}^{odd}(l > 1)$, $E{H}_{lm}^{even}\pm i\ast E{H}_{lm}^{odd}$ and $T{M}_{0m}\pm i\ast T{E}_{0m}$ with non-zero topological charge numbers and m concentric rings.

**Figure 3:**
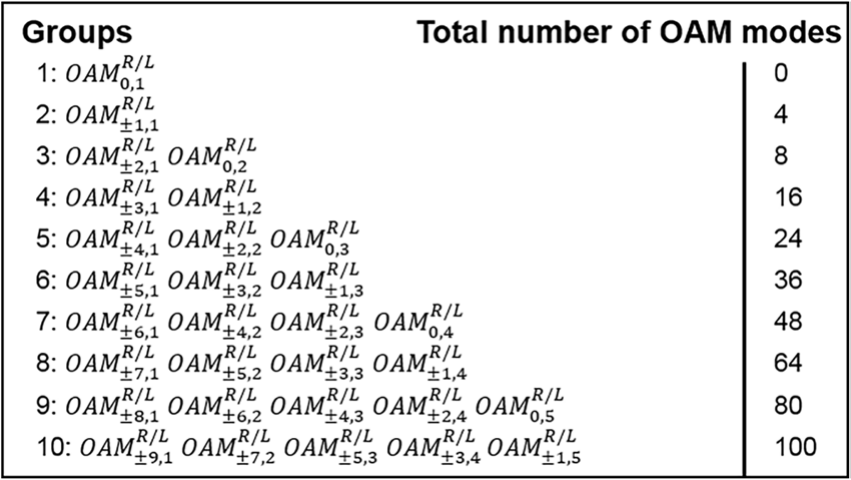
OAM mode groups in conventional graded-index MMF at 1550 nm. Each $OA{M}_{\pm l,m}^{L/R}\,(l > 0)$ is four fold degenerate including polarization and rotation, while $OA{M}_{0,m}^{L/R}$ is two fold degenerate including only polarization.

In Fig. 3, we list all mode groups formed by 30 degenerate OAM modes at 1550 nm that are supported in the conventional graded-index MMF. Actually, the OAM modes in the same group have the same *2* *m* + *l* values. Specifically, the *p* ^*th*^ mode group include *2p* modes with *2* *m* + *l* equal to *p* + *1*. Each $OA{M}_{\pm l,m}^{L/R}\,(l > 0)$ is four fold degenerate including polarization and rotation, while $OA{M}_{0,m}^{L/R}$ is two fold degenerate including only polarization, thus the total number of supported circularly polarized OAM modes is 100, ignoring the other ten OAM modes with zero topological charge number. Those 110 modes are orthogonal with each other and the n_eff_ differences between different groups can be maintained above 10^−4^, which enables low inter-group crosstalk.

### Chromatic dispersion and differential group delay

**Figure 4:**
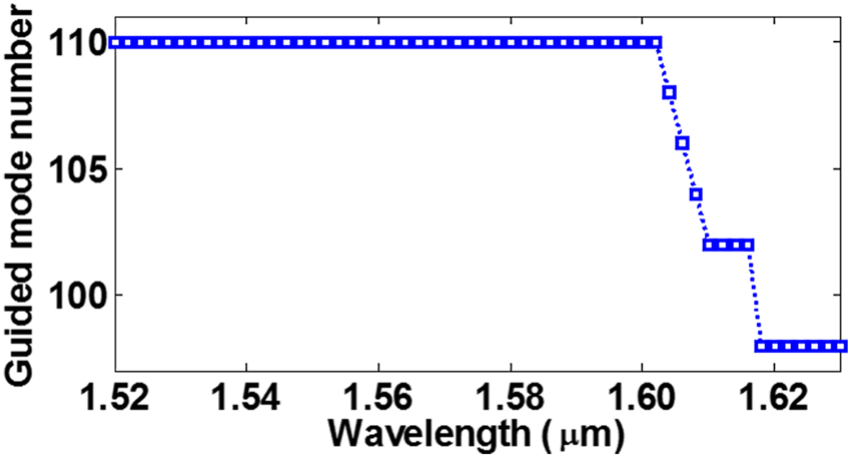
Guided eigenmode number in conventional graded-index MMF versus wavelength.

Conventional graded-index MMF supports hundreds of eigenmodes. Figure 4 shows the calculated guided eigenmode number sweeping wavelength from 1520 to 1630 nm. One can see that the mode number decreases with the increase of the wavelength. In the following calculations, we only present calculated results of the first and last synthesised OAM modes from each group of eigenmodes (typical thirteen OAM modes: *OAM*_0,1_, *OAM*_1,1_, *OAM*_2,1_, *OAM*_3,1_, *OAM*_4,1_, *OAM*_5,1_, *OAM*_6,1_, *OAM*_1,4_, *OAM*_7,1_, *OAM*_0,5_, *OAM*_8,1_, *OAM*_1,5_, *OAM*_3,4_), e.g. *OAM*_0,1_ in the 1^st^ mode group synthesized by *HE*_1,1_ odd/even modes, *OAM*_0,5_ and *OAM*_8,1_ in the 9^th^ mode group synthesized by *HE*_1,5_ odd/even and *HE*_9,1_ odd/even modes, respectively. Here each *OAM*_*l,m*_ mode denotes one of the degenerate $OA{M}_{\pm l,m}^{L/R}$ modes as the degenerate OAM modes have similar dispersion and DGD. Moreover, degenerate OAM modes also have similar effective mode area and nonlinearity, thus we only provide effective mode area and nonlinearity for one of the degenerate $OA{M}_{\pm l,m}^{L/R}$ modes below. Note that the conventional graded-index MMF supports at least 98 modes over 1520 nm to 1630 nm, thus we only take the first 98 modes into consideration.

**Figure 5:**
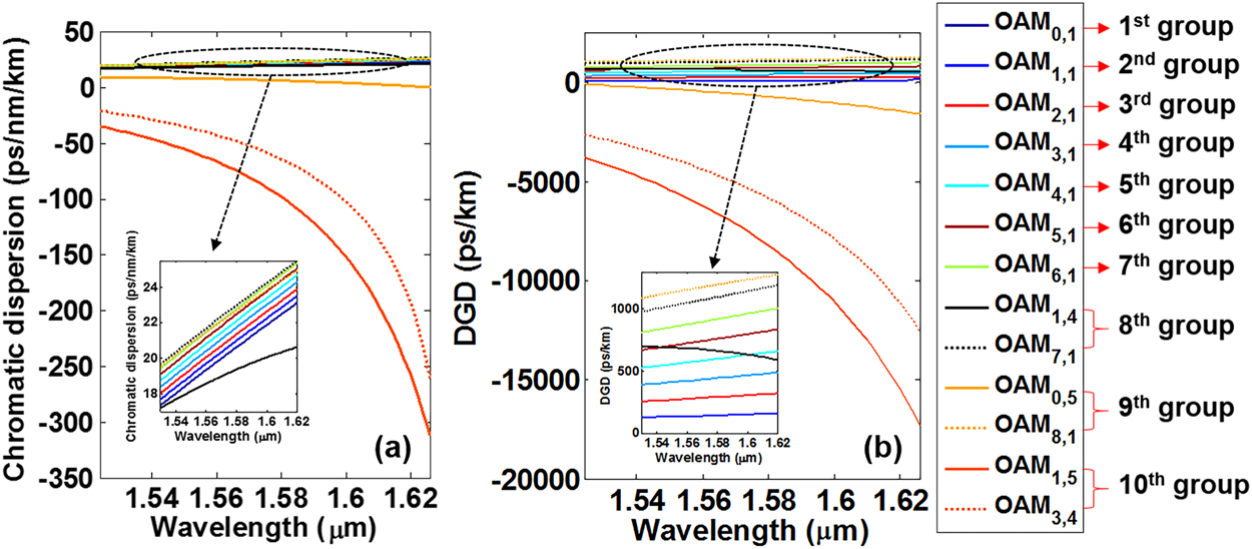
(**a**) Chromatic dispersion and (**b**) differential group delay of typical OAM modes over ten OAM mode groups versus wavelength ranging from 1524 nm to 1626 nm. Each *OAM*_*l*,*m*_ mode denotes one of the degenerate $OA{M}_{\pm l,m}^{L/R}$ modes as the degenerate OAM modes have similar dispersion and differential group delay.

Figure 5 shows the chromatic dispersion and DGD of typical thirteen OAM modes over ten OAM mode groups with the wavelength ranging from 1524 nm to 1626 nm. DGD of *OAM*_*l,m*_ modes is the mode delay between *OAM*_0,1_ mode and *OAM*_*l,m*_ mode, respectively. From Fig. 5(a), one can see that the chromatic dispersion of radially fundamental OAM modes *OAM*_*l*,1_ in the first nine OAM mode groups increases with *l*. The radially fundamental modes in the first nine OAM mode groups have positive chromatic dispersion slightly increasing with the wavelength, while the 10^th^ OAM mode group has negative chromatic dispersion rapidly increasing with the wavelength. In particular, as shown in the inset of Fig. 5(a), one can clearly see that a low and flat chromatic dispersion within (8.9 × 10^−2^, 25.9) ps/nm/km is achieved for the first nine OAM mode groups as the wavelength changes from 1524 to 1626 nm covering the whole C band and L band (1530 to 1625 nm). On the contrary, the 10^th^ OAM mode group has a relatively large and tilted chromatic dispersion within (−312.4, −21.4) ps/nm/km. From Fig. 5(b), one can see that the DGD of radially fundamental OAM modes *OAM*_*l*,1_ in the first nine OAM mode groups increases with *l*. The radially fundamental modes in the first nine OAM mode groups have positive DGD increasing with the wavelength. The DGD of the radially higher-order modes in the first nine OAM mode groups and the 10^th^ OAM mode group decreases with the increase of the wavelength. The radially higher-order mode in the 9^th^ OAM mode group and the 10^th^ OAM mode group have negative DGD. Similarly, a relatively flat DGD within (122.5, 1289.4) ps/km is achieved for the radially fundamental modes in the first nine OAM mode groups, while the 10^th^ OAM mode group has a relatively titled DGD within (−2523.3, −18017.1) ps/km.

**Figure 6:**
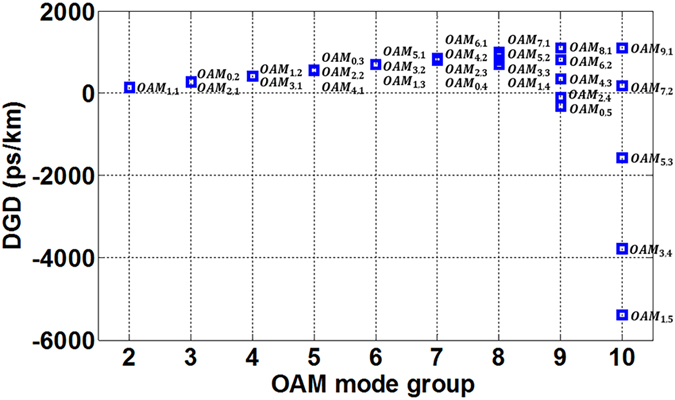
Differential group delay of all 30 degenerate OAM modes at 1550 nm.

Additionally, Fig. 6 shows DGD of all supported degenerate fibre *OAM*_*l,m*_ modes (see also Fig. 3) at 1550 nm. One can clearly see that in the lower OAM mode groups, DGD slightly increases with *m* and decreases with *l*, while in the higher OAM mode groups, DGD rapidly increases with *l* and decreases with *m*. Note that degenerate fibre OAM modes have similar dispersion and DGD. Actually, through similar analyses, we can conclude that the chromatic dispersion of *OAM*_*l,m*_ modes in each mode group increases with *l*, while decreases with *m*.

### Effective mode area and nonlinearity

**Figure 7:**
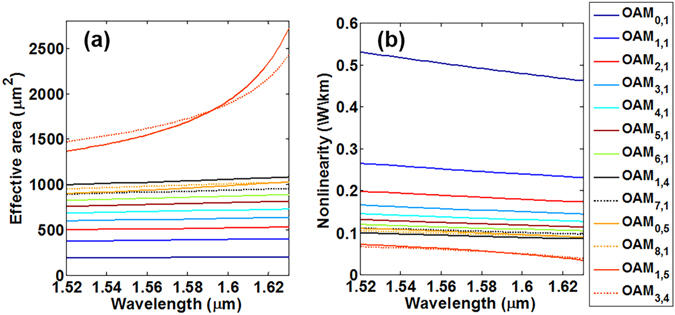
(**a**) Effective mode area and (**b**) nonlinearity of different OAM modes versus wavelength. Each *OAM*_*l*,*m*_ mode denotes one of the degenerate $OA{M}_{\pm l,m}^{L/R}$ modes as the degenerate OAM modes have similar effective mode area and nonlinearity.

Figure 7(a) and (b) show the effective mode area and nonlinearity of typical thirteen OAM modes over the 10 OAM mode groups with the wavelength ranging from 1520 nm to 1630 nm. Note that the fibre nonlinearity is inversely proportional to the effective mode area. One can see that the effective mode area of radially fundamental OAM modes *OAM*_*l*,1_ in the first nine OAM mode groups increases with *l* and wavelength, and the fibre nonlinearity decreases with *l* and wavelength accordingly. Within the whole C band and L band, the conventional graded-index MMF keeps lower nonlinearity. In detail, the first nine OAM mode groups feature flat effective mode area sitting in (186.4546, 1.0816e3) *μm* ^2^, while the 10^th^ OAM mode group has a rapid change of effective mode area sitting in (1.3675e3, 2.7224e3) *μm* ^2^.

**Figure 8:**
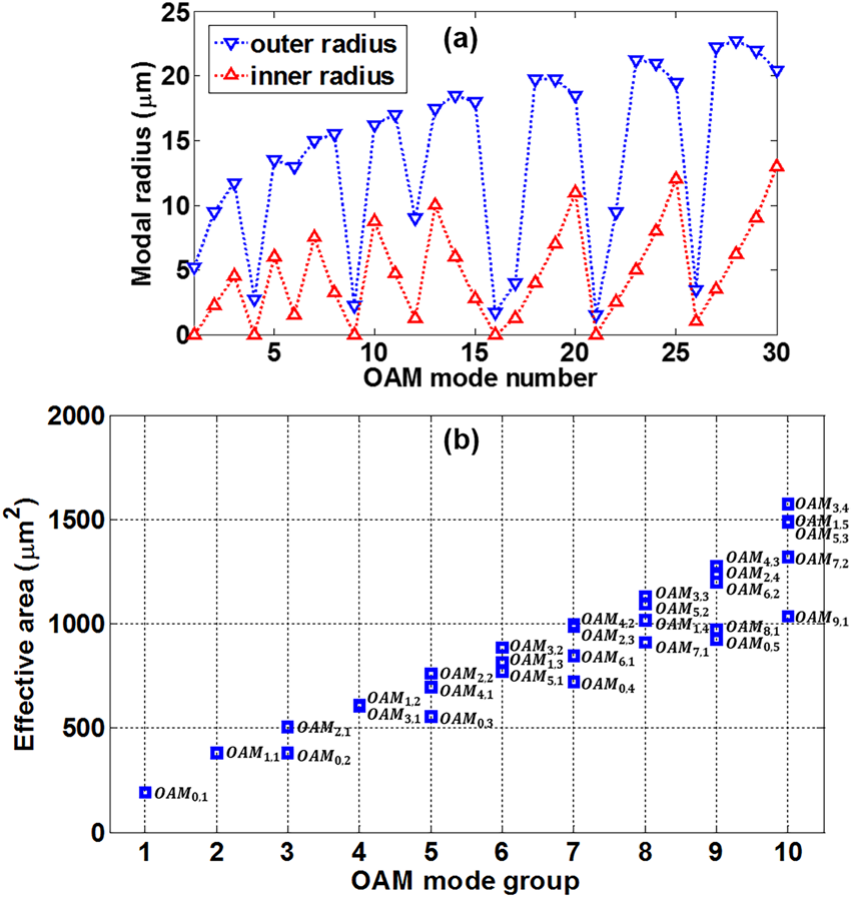
(**a**) The outer 1/*e* ^2^ modal field radius (in blue) and inner radius (in red) and (**b**) effective mode area of all 30 degenerate OAM modes at 1550 nm.

Additionally, Fig. 8(a) shows the outer 1/*e* ^2^ modal field radius (in blue) and inner radius (in red) of all 30 degenerate OAM modes at 1550 nm. The outer modal radius for radially higher-order OAM modes is as large as 22.75 µm which easily leaks to the cladding. Figure 8(b) shows the effective mode area of all supported degenerate fibre *OAM*_*l,m*_ modes at 1550 nm. One can see that the effective mode area of *OAM*_*l,m*_ modes increases with *l* and *m*, that is, for *OAM*_*l,m*_ modes with same *l* (*m*) values, the effective mode area increases with *m* (*l*) values. Generally, the higher OAM mode groups have relatively larger outer modal radius and effective mode area. Note that degenerate OAM modes have similar effective mode area and nonlinearity.

### Tolerance to ellipticity and bending

The intrinsic defects and external perturbations (e.g. stress/strain, squeezing, twisting, bending, heating/temperature) in practical fabrication process and deployment can cause non-ideal fibres. For instance, the stress/strain and squeezing can cause imperfect circularity (i.e. ellipticity) of fibre, which gives rise to the birefringence and resultant polarization mode dispersion of the degenerate fibre eigenmodes. Fibre twisting and bending can also cause birefringence. Those imperfections of non-ideal fibres may cause the difference of propagation constants between the two fibre eigenmodes forming the OAM modes, affect the mode profile and purity of the synthesised OAM modes, and induce OAM modes coupling and crosstalk. Hence, it is of importance to study the effects of non-ideal fibres (ellipticity by stress, fibre bend radius by bending) on the performance of OAM modes.

**Figure 9:**
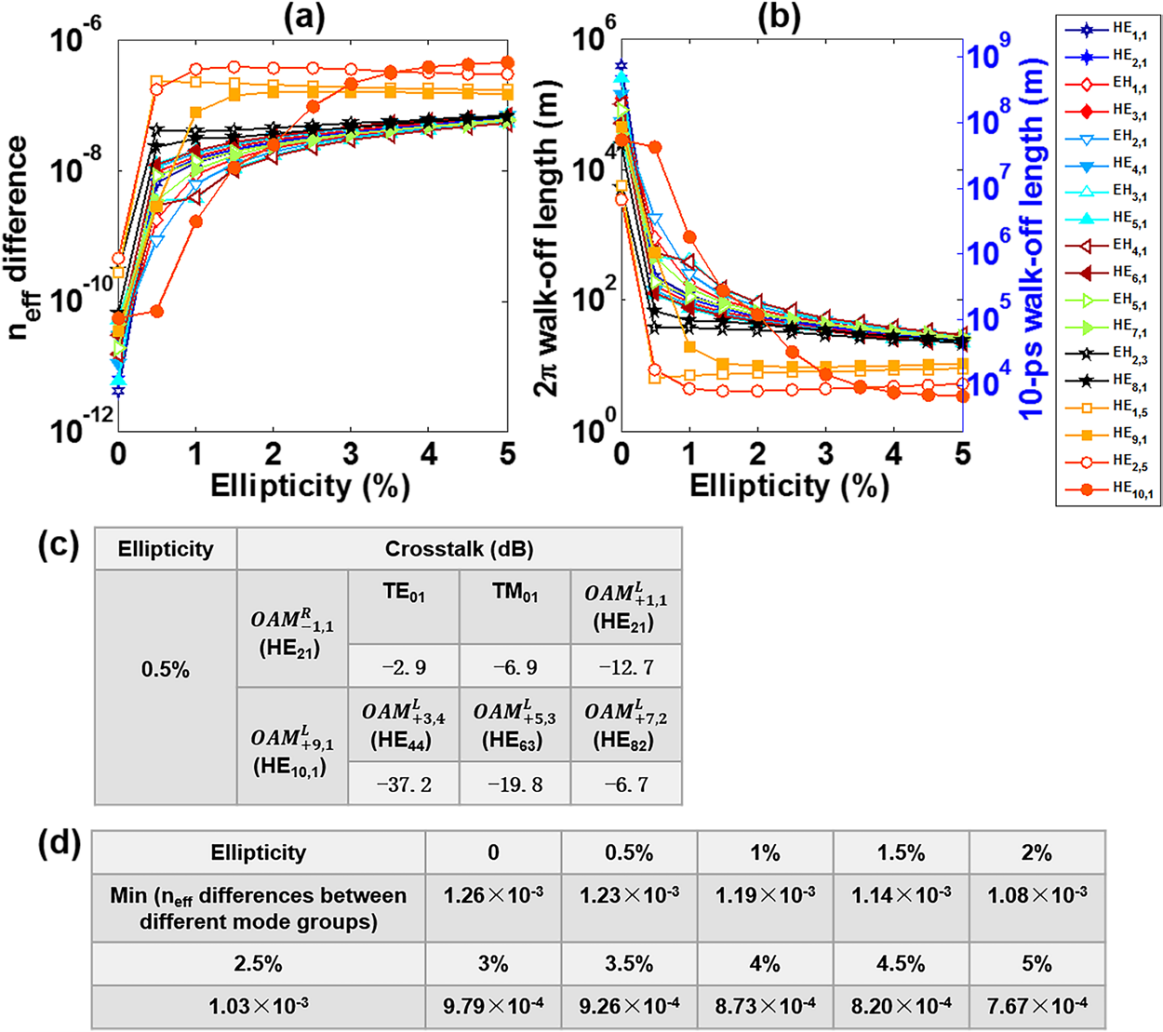
(**a**) n_eff_ difference and (**b**) 2π walk-off length and 10-ps walk-off length between the even and odd fibre eigenmodes versus fibre ellipticity. (**c**) OAM crosstalks for the lowest-order HE_21_ related $OA{M}_{-1,1}^{{\rm{R}}}$ mode and highest-order *HE*_10,1_ related $OA{M}_{+9,1}^{{\rm{L}}}$ mode under an ellipticity of 0.5%. (**d**) Minimum n_eff_ differences between different mode groups with ellipticity varying from 0 to 5%.

We first study the effects of ellipticity on the performance of OAM modes in a conventional graded-index MMF. Figure 9(a) shows the n_eff_ difference between the even and odd fibre eigenmodes which form the OAM modes as a function of the fibre ellipticity. One can see that n_eff_ differences increase with the ellipticity and the growth rate becomes relatively slow with larger ellipticity. When the fibre ellipticity takes 5%, the maximum n_eff_ difference is 4.54 × 10^−7^ for the HE_10,1_ related OAM modes. Additionally, we adopt two parameters 2π walk-off length (*L*_2π_) and 10-ps walk-off length (L_10*ps*_) to characterise the intra-mode walk-off effect upon propagation of the OAM modes resulting from the fibre ellipticity. Figure 9(b) plots the 2π walk-off length and 10-ps walk-off length between the even and odd fibre eigenmodes which form the OAM modes as a function of the fibre ellipticity. Note that the 2π walk-off length and 10-ps walk-off length are inversely proportional to the n_eff_ differences, and the 10-ps walk-off length is around 2000 times longer than the 2π walk-off length at 1550 nm. One can see that the propagation length keeps longer than 6.6 km when the decomposed two fibre eigenmodes have a 10-ps temporal walk off under an ellipticity of 5%. Furthermore, we study the crosstalks between different OAM modes caused by fibre ellipticity. Figure 9(c) presents the calculated OAM crosstalks for the lowest-order ${{\rm{OAM}}}_{-1,1}^{{\rm{R}}}$ mode synthesized by HE_21_ modes and highest-order ${{\rm{OAM}}}_{+9,1}^{{\rm{L}}}$ mode synthesized by HE_10,1_ modes under an ellipticity of 0.5%. Remarkably, the OAM modes coupling and resultant crosstalk only occurs within the same mode group, that is, the crosstalks between different mode groups are negligible. As listed in Fig. 9(c), for the lowest-order ${{\rm{OAM}}}_{-1,1}^{{\rm{R}}}$ mode, the crosstalks to TE_01_, TM_01_ and ${{\rm{OAM}}}_{+1,1}^{{\rm{L}}}$ within the same mode group are −2.9, −6.9 and −12.7 dB, respectively, while less than −40 dB to other channels from different mode groups; for the highest-order ${{\rm{OAM}}}_{+9,1}^{{\rm{L}}}$ mode, the crosstalks to ${{\rm{OAM}}}_{+3,4}^{{\rm{L}}}$, ${{\rm{OAM}}}_{+5,3}^{{\rm{L}}}$ and ${{\rm{OAM}}}_{+7,2}^{{\rm{L}}}$ within the same mode group are −37.2, −19.8 and −6.7 dB, respectively, while less than −40 dB to other channels within the same mode group or from different mode groups. In particular, we calculate the minimum n_eff_ differences between different mode groups with ellipticity varying from 0 to 5%, as listed in Fig. 9(d). Although the minimum n_eff_ difference between different mode groups decreases with the increase of ellipticity, it still keeps larger than 7.67 × 10^−4^ even under an ellipticity of 5%, indicating low-level crosstalk between different mode groups.

**Figure 10:**
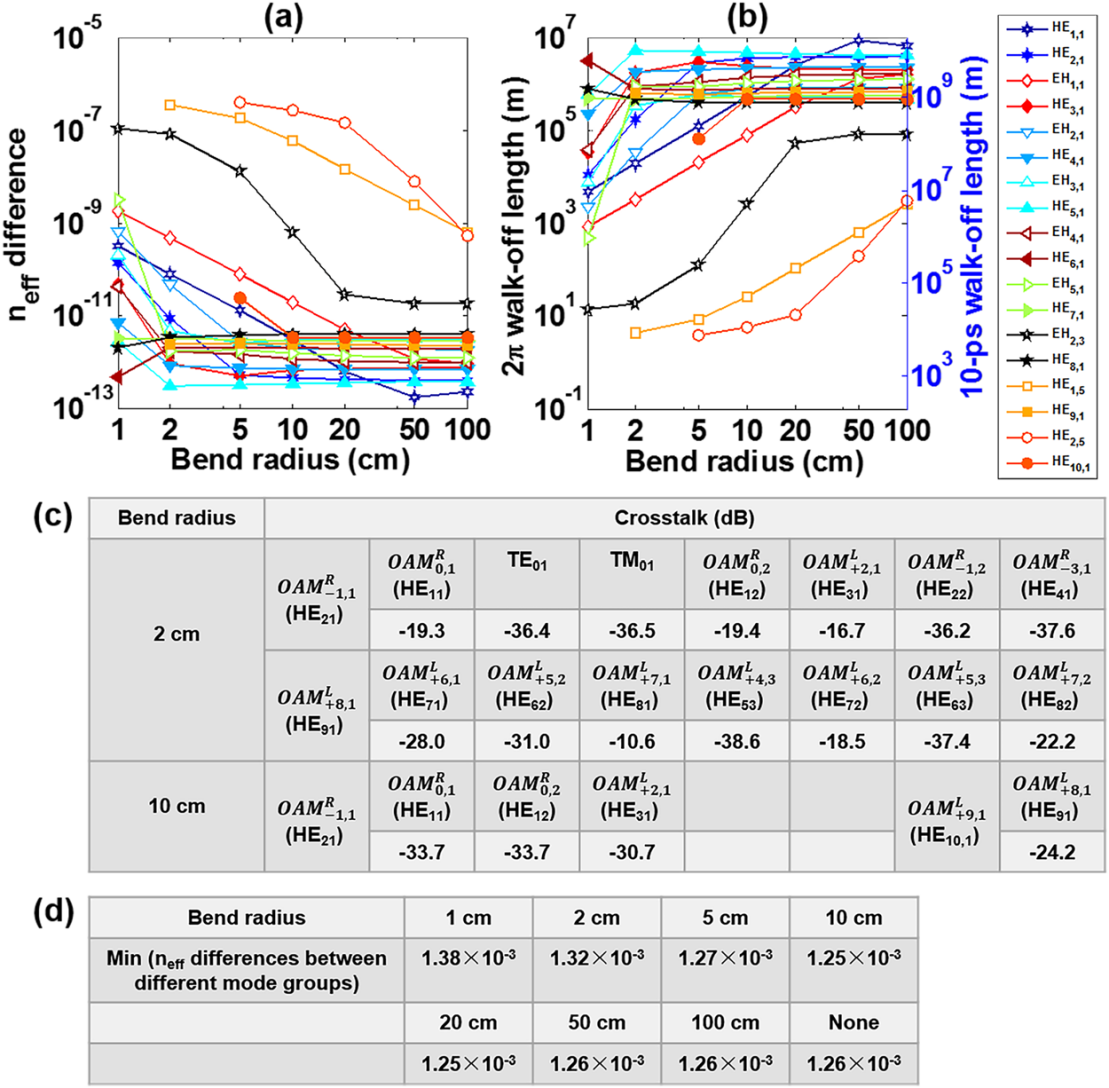
(**a**) n_eff_ difference and (**b**) 2π walk-off length and 10-ps walk-off length between the even and odd fibre eigenmodes versus fibre bend radius. (**c**) OAM crosstalks for the lowest-order HE_21_ related ${{\rm{OAM}}}_{-1,1}^{{\rm{R}}}$ mode and highest-order HE_91_ related ${{\rm{OAM}}}_{+8,1}^{{\rm{L}}}$ mode or HE_10,1_ related ${{\rm{OAM}}}_{+9,1}^{{\rm{L}}}$ mode under fibre bend radii of 2 cm and 10 cm, respectively. (**d**) Minimum n_eff_ differences between different mode groups with bend radius varying from 1 cm to 100 cm. ‘None’ means no bending.

We then discuss the characteristics of OAM modes under different fibre bend radius. Note that the higher-order modes leak out from the core into the cladding with the decrease of bend radius. As a result, the number of supported eigenmodes reduces to 90 and 72 under fibre bend radii of 2 cm and 1 cm, respectively. Figure 10(a) shows the n_eff_ difference between the even and odd fibre eigenmodes which form the OAM modes as a function of the fibre bend radius. One can see that n_eff_ differences increase with the decrease of fibre bend radius. The azimuthally higher-order radially fundamental OAM modes show stronger tolerance to the bend radius variation, while the radially higher-order OAM modes show larger n_eff_ differences. When the bend radius is 1 cm, the maximum n_eff_ difference is 1.11 × 10^−7^ for the EH_23_ related OAM modes. Figure 10(b) plots the $2{\rm{\pi }}$ walk-off length and 10-ps walk-off length between the even and odd fibre eigenmodes which form the OAM modes as a function of fibre bend radius. The minimum 10-ps walk-off length is 26.9 km with 1 cm bend radius. Figure 10(c) presents the calculated OAM crosstalks for the lowest-order HE_21_ related ${{\rm{OAM}}}_{-1,1}^{{\rm{R}}}$ mode and highest-order HE_91_ related ${{\rm{OAM}}}_{+8,1}^{{\rm{L}}}$ mode or HE_10,1_ related ${{\rm{OAM}}}_{+9,1}^{{\rm{L}}}$ mode under fibre bend radii of 2 cm and 10 cm, respectively. When the bend radius takes 2 cm, the crosstalks for HE_21_ and HE_91_ related OAM modes are less than −16.7 dB and −10.6 dB, respectively, while the crosstalks for HE_21_ and HE_10,1_ related OAM modes with 10 cm bend radius are less than −30.7 dB and −24.2 dB, respectively. It is found that the lower-order OAM modes show relatively low-level crosstalk. Moreover, we calculate the minimum n_eff_ differences between different mode groups with bend radius varying from 1 cm to 100 cm, as listed in Fig. 10(d). It is noted that the minimum n_eff_ difference between different mode groups keeps larger than 1.25 × 10^−3^ when decreasing the bend radius.

### Robustness against fibre perturbations

In addition to ellipticity and bending, the heating or temperature variation can cause slight change of geometric parameters and material properties of fibre, i.e. the practically fabricated MMF might vary slightly in the core size, index contrast between the core and cladding, the graded index profile and so on. Thus we further address the robustness against fibre perturbations including the fibre core size, the core index and the distorted fibre index profile.

**Figure 11:**
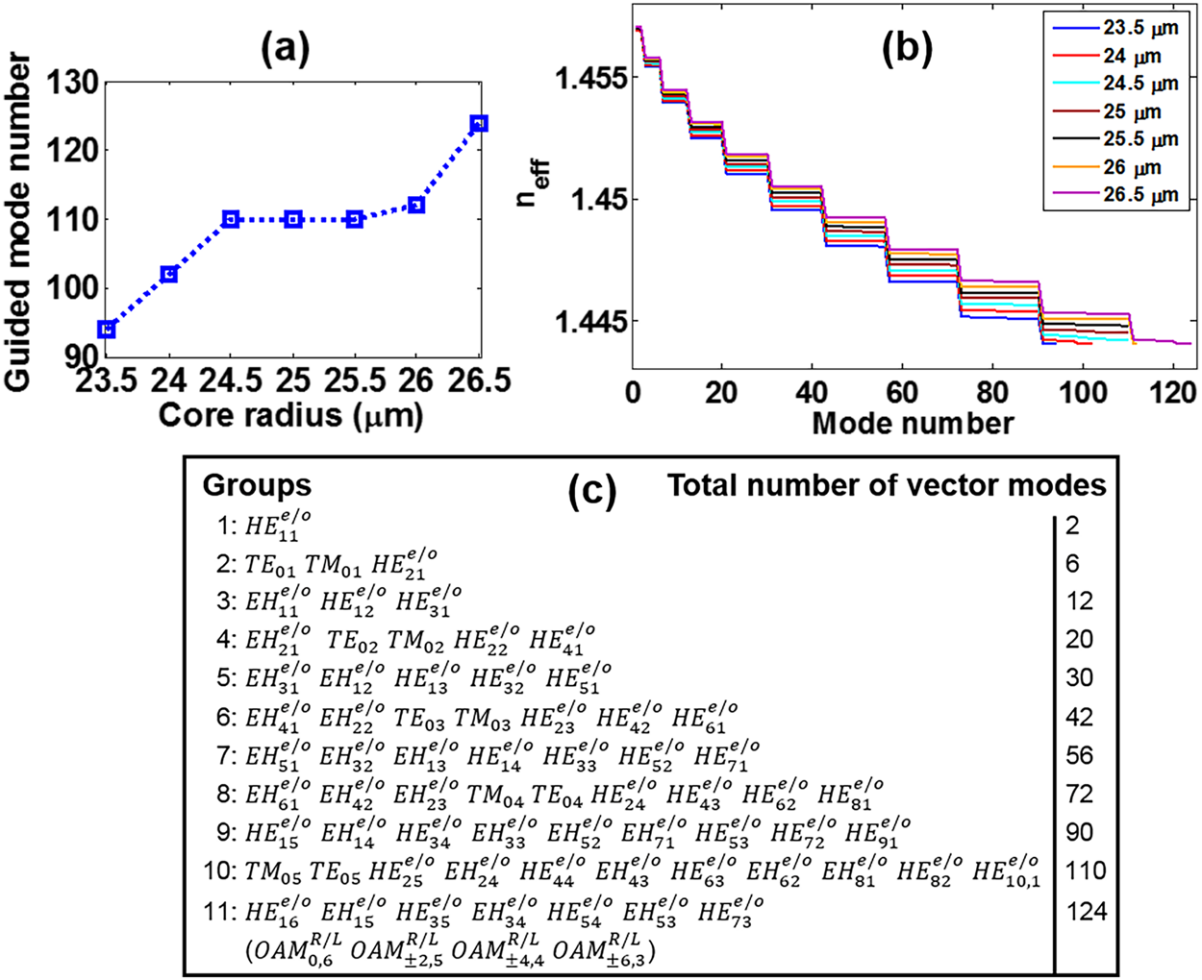
(**a**) Guided mode number and (**b**) n_eff_ distribution versus fibre core radius. (**c**) All 124 fibre modes when the fibre core radius takes 26.5 µm.

The fibre core diameter of the actual drawn MMF is assumed to have a deviation of 25 ± 2.5 µm. By sweeping the fibre core radius from 23.5 µm to 26.5 µm (with 0.5 µm spacing) at 1550 nm, the calculated guided eigenmode number and n_eff_ distribution are shown in Fig. 11(a) and (b). With the increase of fibre core radius, the n_eff_ of the same order fibre modes increase accordingly and more fibre eigenmodes are guided in the MMF. Moreover, within a small radius deviation of 25 ± 0.5 µm, the MMF keeps supporting 110 eigenmodes. When the fibre core radius becomes 26.5 µm, Fig. 11(c) lists all 124 fibre cylindrical vector modes supported including additional 14 eigenmodes (or OAM modes) in the last group compared with a 25-µm core radius MMF. Note that in the same mode groups, the mode order may change with different fibre parameters, such as the fibre core size, the core index and the perturbed fibre profiles.

**Figure 12:**
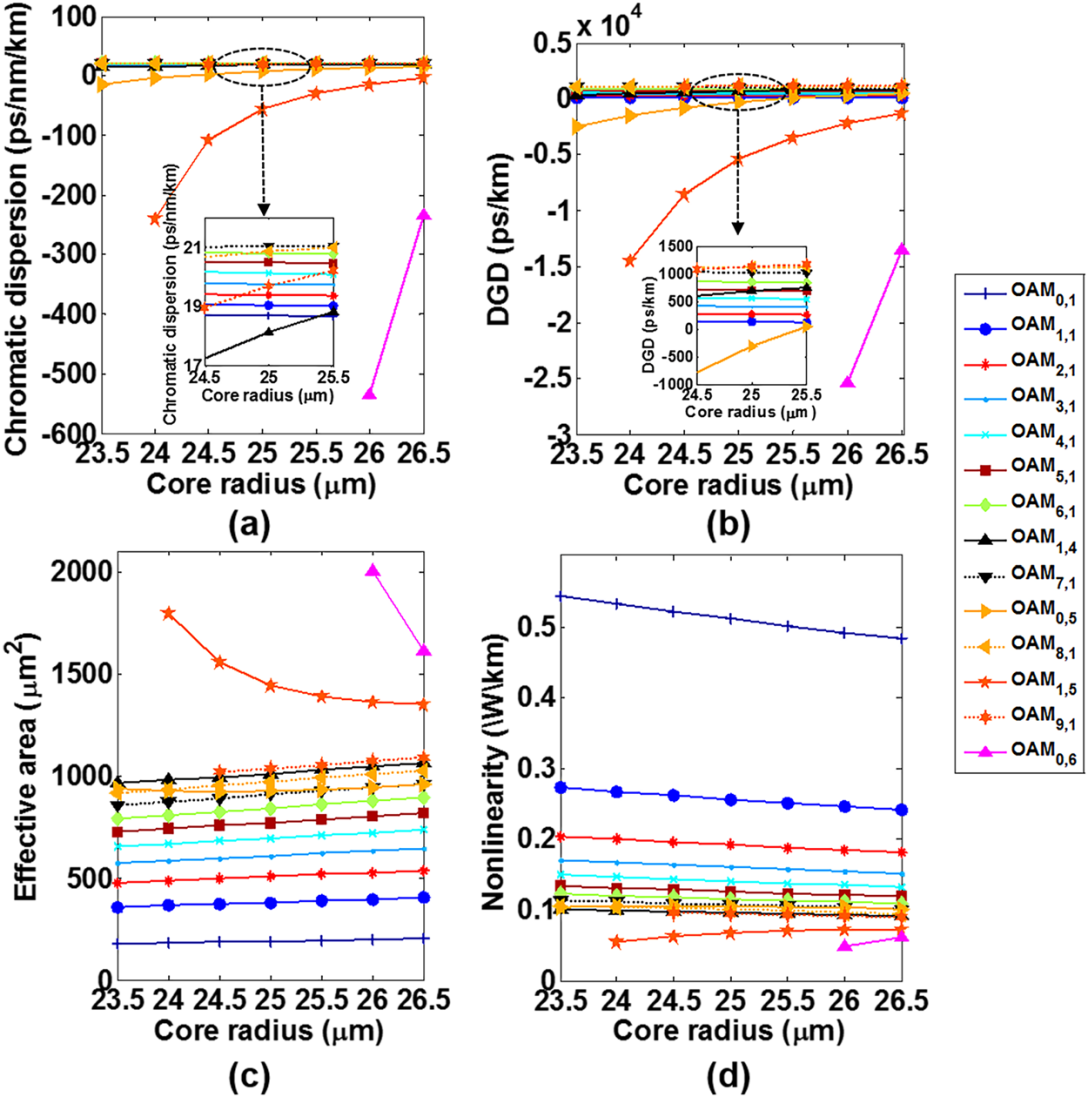
(**a**) Chromatic dispersion, (**b**) differential group delay, (**c**) effective mode area, and (**d**) nonlinearity coefficient for the typical fourteen OAM modes versus fibre core radius.

Figure 12 shows the calculated chromatic dispersion, DGD, effective mode area, and nonlinearity coefficient for the typical fourteen OAM modes discussed above versus fibre core radius. One can clearly see that the lower-order OAM modes in radial direction feature stronger tolerance to fibre core radius variation. For the higher-order modes in radial direction (OAM_0,5_, OAM_1,5_ and OAM_0,6_), the chromatic dispersion, DGD and nonlinearity coefficient increase with fibre core size, while the effective mode area decreases with the increase of fibre core size.

**Figure 13:**
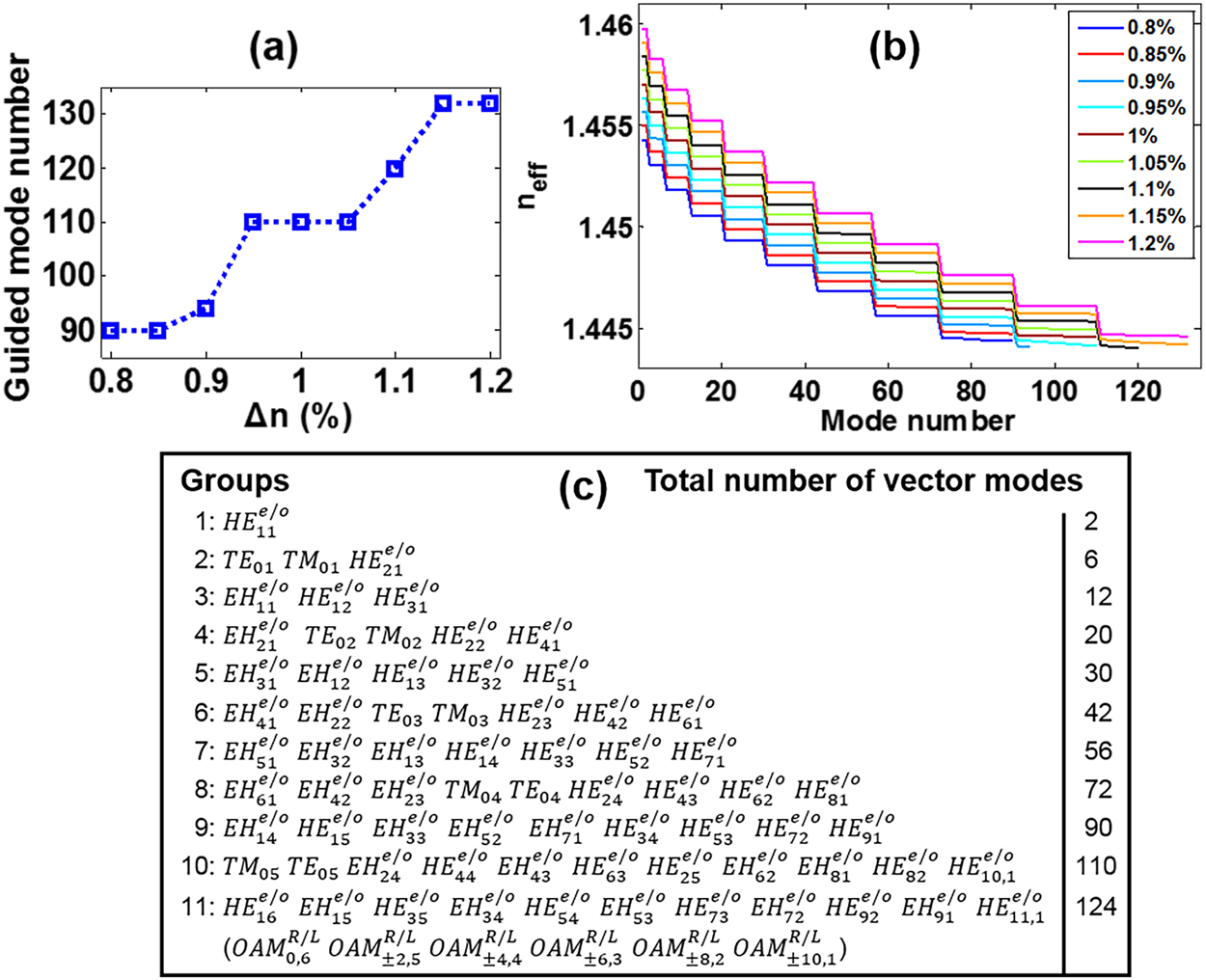
(**a**) Guided mode number and (**b**) n_eff_ distribution versus fibre relative refractive index difference. (**c**) All 132 fibre modes when the fibre relative refractive index difference is 1.2%.

The fibre numerical aperture of the actual drawn MMF is assumed to have a deviation of 0.2 ± 0.015 (with fibre relative refractive index difference (Δn) changing from 0.82% to 1.1%). Similarly, we sweep the fibre relative refractive index difference from 0.8% to 1.2% (with 0.05% spacing) with fibre cladding index fixed to 1.4440 at 1550 nm. Figure 13(a) shows the calculated guided eigenmode number which keeps supporting 110 eigenmodes with the core index difference deviation of 1% ± 0.5%. The detailed n_eff_ distributions are shown in Fig. 13(b). When the fibre relative refractive index difference becomes 1.2%, 132 modes including additional 22 eigenmodes (or OAM modes) in the 11^th^ group compared with a 1% relative refractive index difference MMF are supported as shown in Fig. 13(c).

**Figure 14:**
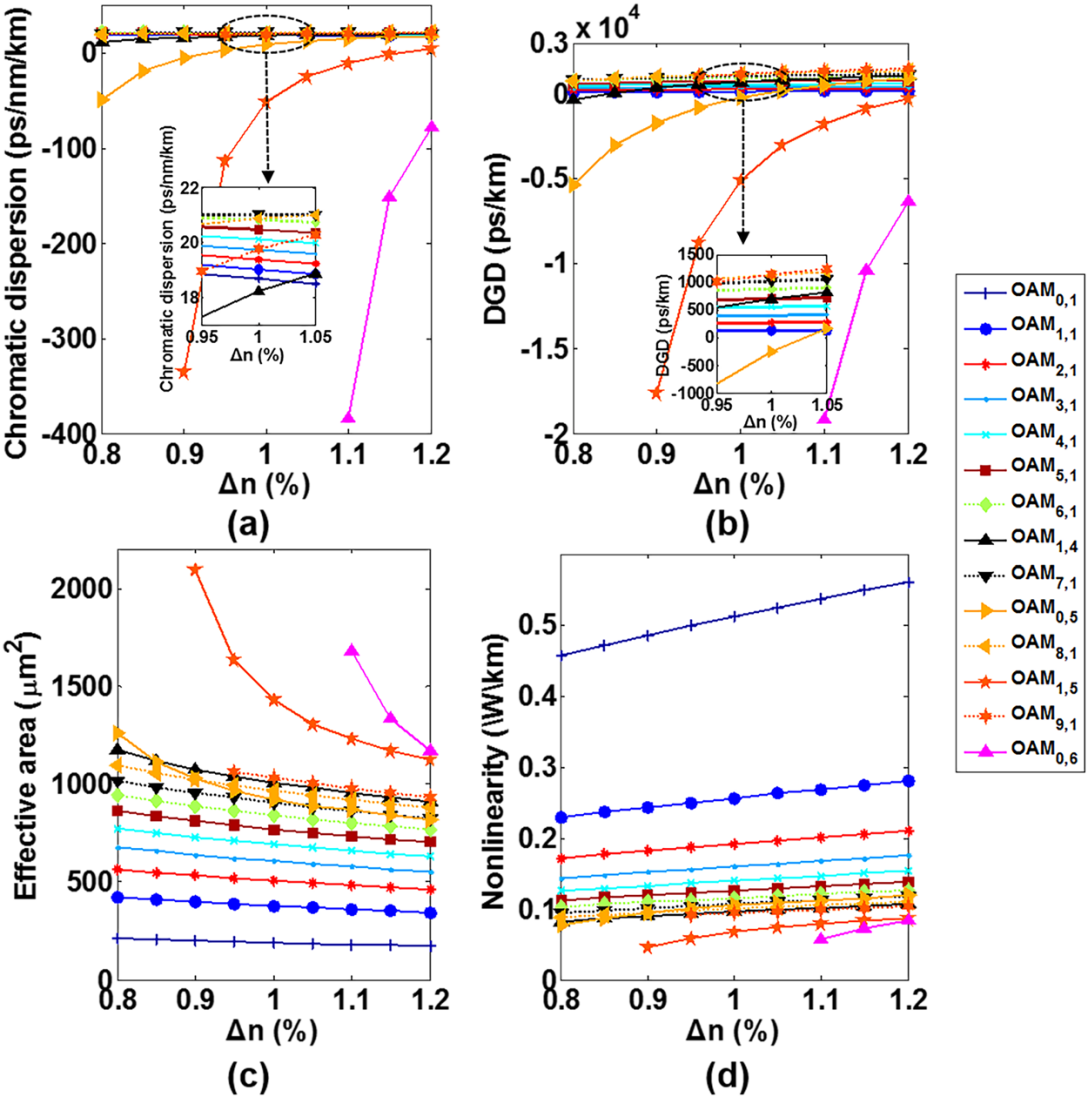
(**a**) Chromatic dispersion, (**b**) differential group delay, (**c**) effective mode area, and (**d**) nonlinearity coefficient for the typical fourteen OAM modes versus fibre relative refractive index difference.

Figure 14 shows the calculated chromatic dispersion, DGD, effective mode area, and nonlinearity coefficient for the typical fourteen OAM modes as a function of the fibre relative refractive index difference. Similar to the core size perturbation effects, the lower-order OAM modes in radial direction feature larger tolerance to fibre core index variation, while the chromatic dispersion, DGD and nonlinearity coefficient of the higher-order modes in radial direction increase with fibre relative refractive index difference.

**Figure 15:**
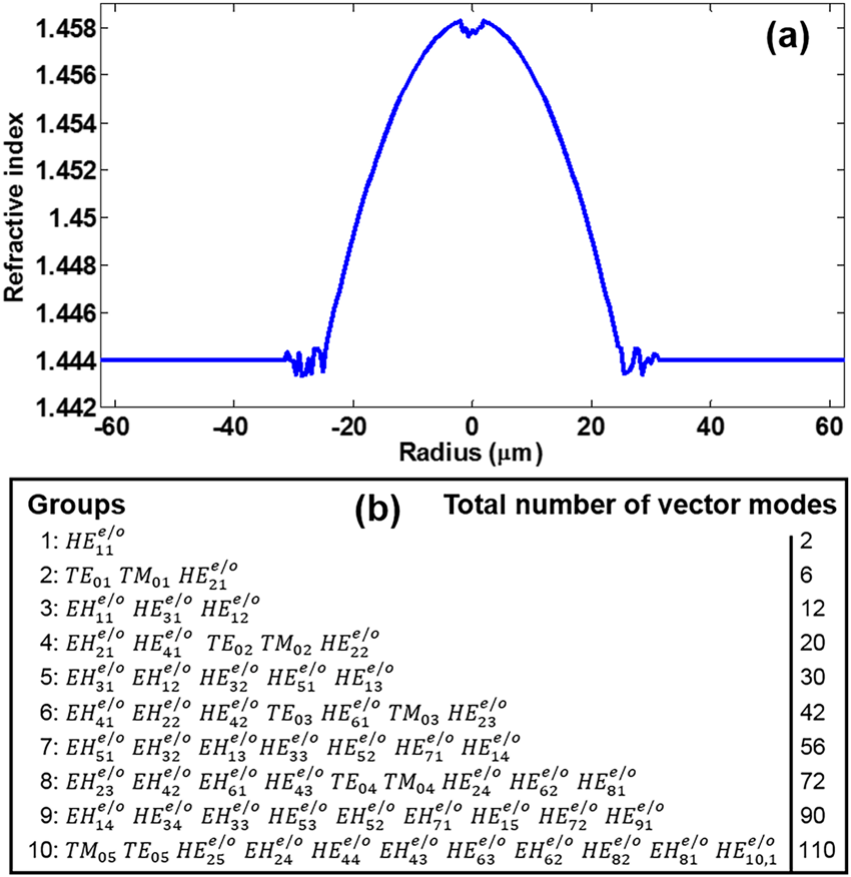
(**a**) Simulated refractive index profile distorted by random ripples with ripple amplitude of 0.5‰. (**b**) All 110 fibre modes when the ripple amplitude is 0.5‰.

Due to the limitations of modern fibre manufacture technology, the actual drawn MMF might have slightly distorted square law profile, typically with a central index dip and the refractive index ripple near the core-cladding boundary^47^. Here we accordingly simulate a refractive index profile that is distorted by random ripples in the fibre core center and core-cladding boundary as shown in Fig. 15(a). By sweeping the ripple amplitude from 0 to 1‰ (with 0.1‰ spacing), we analyse the fibre robustness against the distorted fibre index profile. Note that in Fig. 15(a), the ripple amplitude is 0.5‰, that is, the maximum index perturbation has 0.5‰ index difference with the idealized index at the same position. The supported eigenmodes number keeps 110 and the n_eff_ distributions are almost the same. Figure 15(b) lists all the 110 eigenmodes when the ripple amplitude is 0.5‰.

**Figure 16:**
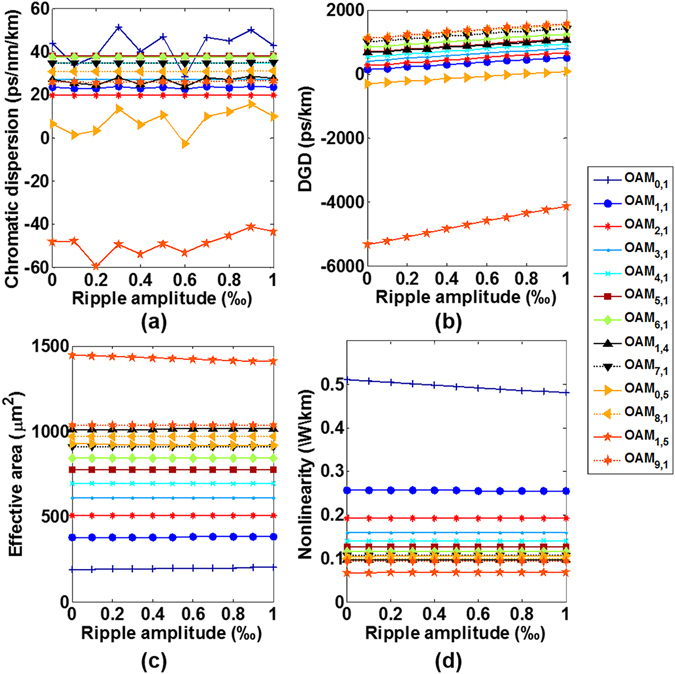
(**a**) Chromatic dispersion, (**b**) differential group delay, (**c**) effective mode area, and (**d**) nonlinearity coefficient for the typical thirteen OAM modes versus ripple amplitude.

Figure 16 shows the calculated chromatic dispersion, DGD, effective mode area, and nonlinearity coefficient for the typical thirteen OAM modes as a function of the ripple amplitude. One can see that with the increase of ripple amplitude, the chromatic dispersion of most of OAM modes keeps almost constant, while OAM_0,1_, OAM_1,4_, OAM_0,5_ and OAM_1,5_ vary in a range less than 23.1, 4.9, 18.4 and 18.5 ps/nm/km, respectively. The DGD increases with the ripple amplitude, showing reduced fibre bandwidth when increasing the ripple amplitude. The effective mode area and nonlinearity coefficient vary slightly with the increase of ripple amplitude.

## Discussions

OAM modes featuring helical phase fronts have recently seen emerging applications of optical communications in free space and specially designed fibres. It is always believed that specialty fibres are preferable to support OAM modes. However, the most widely deployed or commercially available fibres are SMF and MMF. Conventional MMFs are widespread for short-reach optical interconnect applications because of their relaxed connector tolerances and efficient coupling to low-cost laser sources. Many present and future optical communication links for enterprise and data centre applications are based on MMFs. Fundamentally, the standard SMF does not support OAM modes at 1550 nm. It is valuable to exploit conventional MMF for OAM communications.

In summary, we give a comprehensive characterisation of OAM modes in the conventional MMF. We study the synthesis of OAM modes, effective refractive index, number of OAM modes, division of OAM mode groups, chromatic dispersion, DGD, effective mode area, and nonlinearity. We discuss in detail the effects of non-ideal fibres (variation of core size, refractive index and index profile by heating/temperature, ellipticity by stress/strain, fibre bend radius by bending) on the performance of OAM modes. The n_eff_ differences, 2π walk-off length, 10-ps walk-off length, OAM crosstalks, and minimum n_eff_ differences between different mode groups are analysed. The conventional MMF shows favorable tolerances to heating/temperature/stress/strain/bending induced fibre perturbations, indicating its potential robustness even used in practical applications.

The obtained results provide detailed theoretical understanding on OAM modes supported in the conventional MMF and show the feasibility to use the conventional MMF for OAM multiplexing communications. The OAM modes guided in the conventional MMF can be divided into multiple groups. Two OAM multiplexing approaches could be considered. First, one can employ different OAM mode groups to carry different data information channels. Different OAM mode groups with relatively large effective refractive index difference have low-level inter-group crosstalk, showing possible OAM mode group multiplexing communications. Second, one can also use different OAM modes to carry different data information. In the same OAM mode group with relatively large intra-group crosstalk, small-scale multiple-input multiple-output digital signal processing (MIMO-DSP) technique could be used to mitigate the mode crosstalk. Hence, small-scale MIMO-DSP assisted intra-group OAM modes multiplexing combined with crosstalk-free inter-group OAM modes multiplexing can be considered to facilitate the OAM multiplexing communications using a conventional MMF. With future improvement, experimental demonstrations on OAM modes multiplexing communications in the conventional MMF are under way. One challenge is the perfect excitation of OAM modes in the conventional MMF. Previous work has shown the excitation of OAM modes in specially designed fibres via perfect OAM beams^48^, which could provide an effective approach to facilitating perfect excitation of OAM modes in the conventional MMF.

It is of great interest to directly employ commercially available MMF but not specially designed and fabricated fibre with increased complexity for OAM communications. The presented work may open up new perspectives to more extensive OAM related applications using already existing conventional MMF.

## Methods

### Chromatic dispersion, differential group delay, effective mode area, and nonlinearity

In the characterisation of OAM modes in conventional graded-index MMF (OM3 fibre), we calculate the chromatic dispersion (*D*_*λ*_), differential group delay (DGD), effective mode area (A_eff_), and nonlinearity coefficient (γ) for the first and last synthesized OAM modes in each OAM mode group over a wide wavelength ranging from 1520 to 1630 nm covering the whole C band and L band (1530 to 1625 nm). The chromatic dispersion, mode delay, effective mode area and nonlinearity coefficient associated with the *p* th eigenmode are expressed as follows^49, 50^.

1 $${D}_{\lambda }=-\lambda /c\cdot {d}^{2}{n}_{eff}/d{\lambda }^{2}$$

2 $${\tau }_{{\rm{p}}}=-{\lambda }^{2}/2\pi c\cdot d{\beta }_{p}/d\lambda $$

3 $${A}_{eff,p}(\lambda )={[\int d{{\boldsymbol{r}}}_{\perp }{I}_{p}({{\boldsymbol{r}}}_{\perp })]}^{2}/\int d{{\boldsymbol{r}}}_{\perp }{I}_{p}^{2}({{\boldsymbol{r}}}_{\perp })$$

4 $$\gamma ={\rm{2}}\pi {n}_{2}/(\lambda {A}_{eff})$$

where *c* is the light velocity in vacuum, *λ* is the wavelength in vacuum, n_eff_ is the effective refractive index, β is the phase constant of the fibre eigenmode, $\,{I}_{p}({r}_{\perp })$ is the intensity distribution, and *n*_2_ = 2.4 × 10^−20^ *m* ^2^/*W* is the nonlinear refractive index of pure *SiO*_2_, respectively.

### Convergence

**Figure 17:**
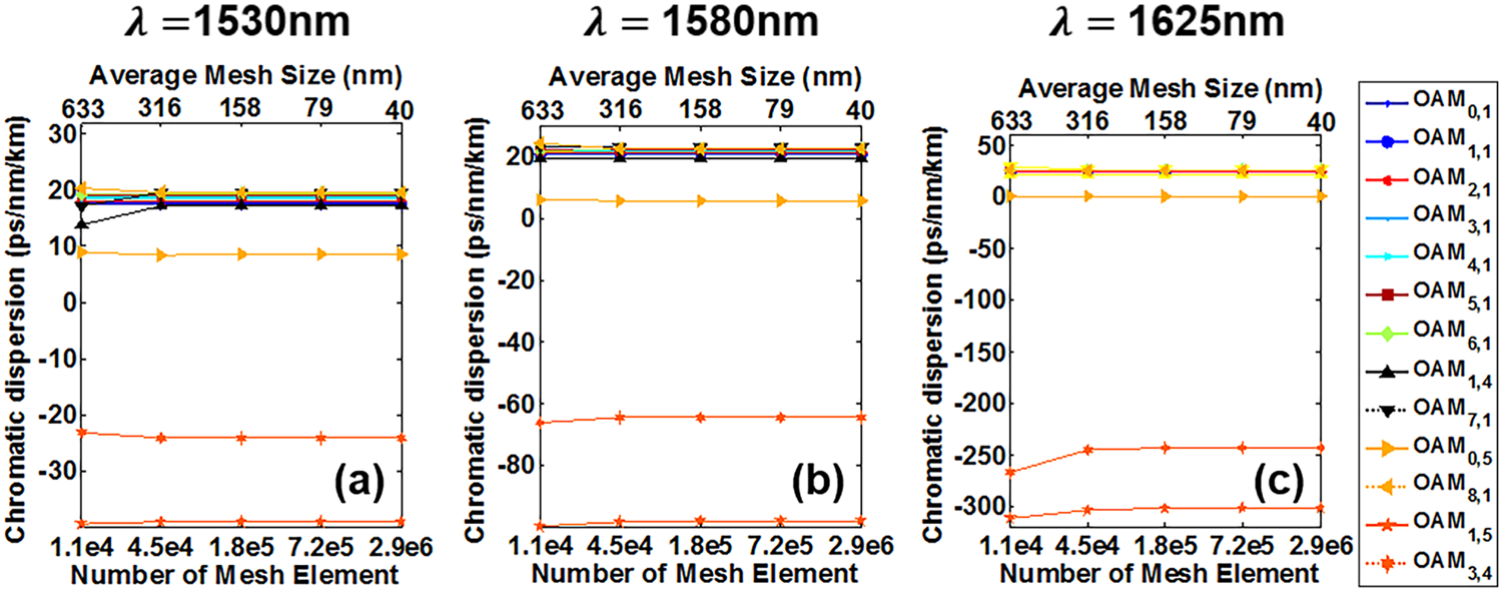
Chromatic dispersion versus number of mesh element and average mesh size for different OAM modes at 1530 nm, 1580 nm and 1625 nm, respectively.

In order to ensure accurate calculations of all higher-order modes, the mesh element in simulations should be finer. With the increase of the number of the mesh element, the precision of the computational results would be improved, while the complexity of the computation process also increases accordingly. Here we verify the convergence of the calculated chromatic dispersion values under different number of mesh element in the fibre core region. Figure 17 shows chromatic dispersion values of OAM modes across the C band and L band at 1530 nm, 1580 nm and 1625 nm with the number of mesh element increasing from 1.1e4 to 2.9e6, that is, the average mesh size decreasing from 633 nm to 40 nm. When the number of mesh element is greater than 1.4e4, the chromatic dispersion of all OAM modes keeps almost constant. Hence, the mesh element used to calculate chromatic dispersion has a number of 1.4e4 and the average mesh size is 158 nm.

**Figure 18:**
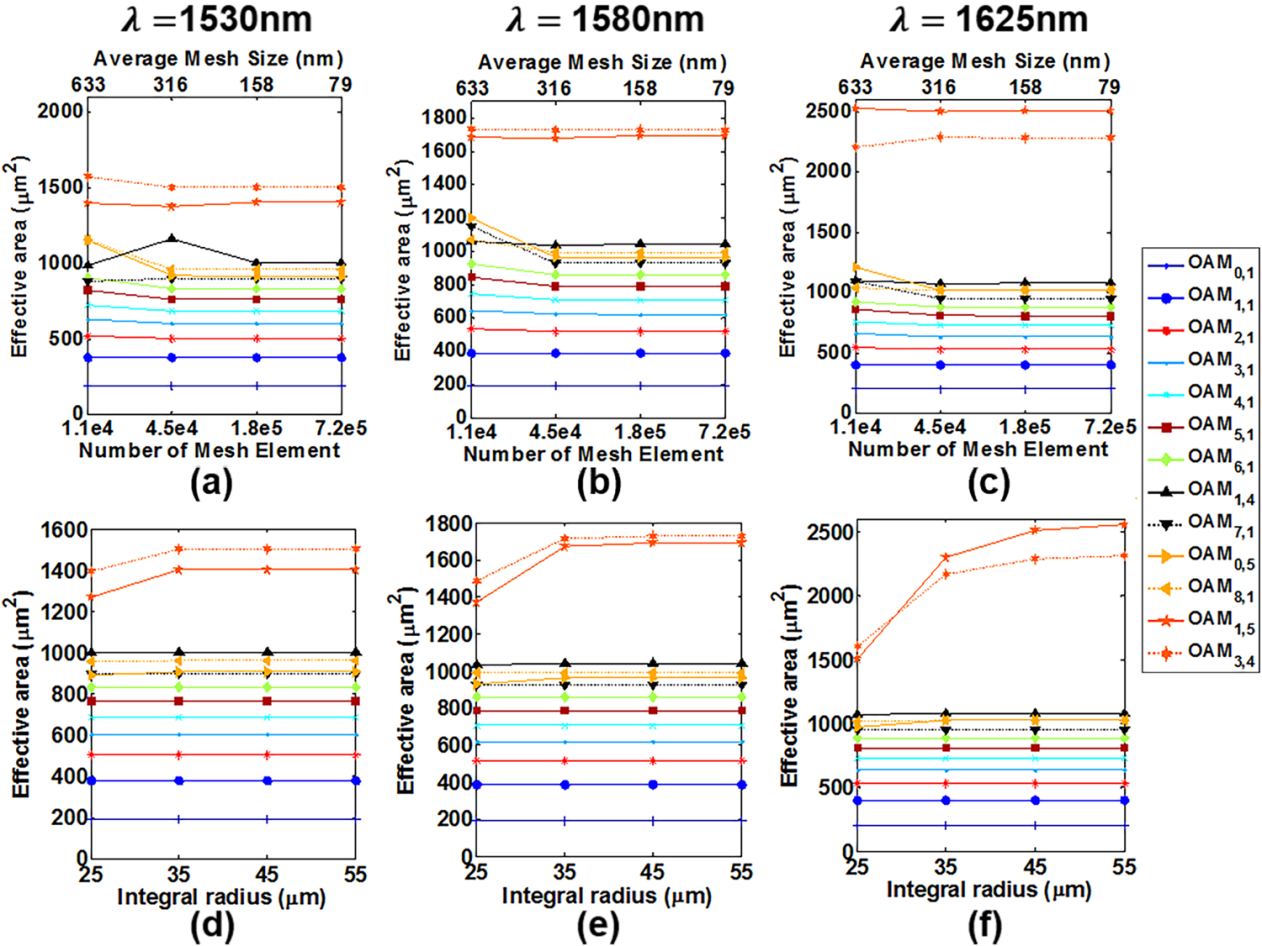
(**a**–**c**) Effective mode area versus number of mesh element/average mesh size across the C band and L band. The integral radius is 45 µm. (**d**–**f**) Effective mode area versus integral radius across the C band and L band. The average mesh size is 158 nm. (**a**,**d**) 1530 m; (**b**,**e**) 1580 nm. (**c**,**f**) 1625 nm.

Similarly, in order to ensure accurate calculations of all higher-order modes, we verify the convergence of the calculated effective mode area values under different integral region and number of mesh element in the fibre core region. Here we still show typical thirteen OAM modes over 10 OAM mode groups for analyses. Figure 18(a–c) show effective mode area values of OAM modes across the C band and L band at 1530 nm, 1580 nm and 1625 nm with the number of mesh element increasing from 1.1e4 to 7.2e5, that is, the average mesh size decreasing from 633 nm to 79 nm and the integral radius remaining 45 µm. Figure 18(d–f) sweep the integral radius changing from 25 µm to 55 µm and the number of mesh element remains 1.1e4. According to the results shown in Fig. 18, we eventually employ the average mesh size of 158 nm with 1.4e4 mesh element and integral radius of 45 µm for simulations of effective mode area and nonlinearity.

### Walk-off length

2π walk-off length (*L*_2π_) and 10-ps walk-off length (*L*_10*ps*_) denote the propagation length when the even and odd fibre eigenmodes walk off to each other with a 2π relative phase shift or have a 10-ps temporal walk-off.

The expressions are written by^36^

5 $${L}_{2\pi }=\frac{\lambda }{{n}_{eff}^{even}-{n}_{eff}^{odd}}$$

6 $${L}_{10ps}=\frac{c\times {\rm{\Delta }}t}{{n}_{eff}^{even}-{n}_{eff}^{odd}}$$

where λ and *c* are the wavelength and light velocity in vacuum, ${n}_{eff}^{even}$ and ${n}_{eff}^{odd}$ are effective refractive index of the even and odd fibre eigenmodes, and Δt = 10 ps is the temporal walk off time.

### OAM crosstalks

OAM crosstalks, also characterised by OAM spectra or OAM charge weight distributions, are expressed by 10log_10_|*C*_*i*_|^2^, with *C*_*i*_ written by^51^

7 $${C}_{i}=\frac{\iint e(x,y){\psi }_{i}^{\ast }(x,y)dxdy}{({\iint }_{\infty }e(x,y){e}^{\ast }(x,y){\iint }_{\infty }{\psi }_{i}(x,y){\psi }_{i}^{\ast }(x,y)dxdy{)}^{1/2}}$$

where *ψ*_*i*_(*x*, *y*) and *e*(*x*, *y*) are the electric field distributions of the synthesised circular OAM modes in the ideal fibre and the perturbed fibre with ellipticity or bending, respectively.
